# Impact of legal regulation on elective cesarean sections in a secondary complexity maternity hospital in São Paulo state

**DOI:** 10.61622/rbgo/2025rbgo50

**Published:** 2025-07-15

**Authors:** Giulia Lopes Corte Mainardi, Vinicius Aniceto, João Vitor Zaniboni de Assumpção, Caio Antonio de Campos Prado, Ana Carolina Tagliatti Zani Mantovi, Elaine Christine Dantas Moisés

**Affiliations:** 1 Universidade de São Paulo Faculdade de Medicina de Ribeirão Preto Ribeirão Preto SP Brazil Faculdade de Medicina de Ribeirão Preto, Universidade de São Paulo, Ribeirão Preto, SP, Brazil.; 2 Hospital das Clínicas da Faculdade de Medicina de Ribeirão Preto Ribeirão Preto SP Brazil Hospital das Clínicas da Faculdade de Medicina de Ribeirão Preto, Ribeirão Preto, SP, Brazil.; 3 Centro de Referência da Saúde da Mulher de Ribeirão Preto Ribeirão Preto SP Brazil Centro de Referência da Saúde da Mulher de Ribeirão Preto, Ribeirão Preto, SP, Brazil.

**Keywords:** Vaginal birth, Cesarean section, Pregnancy, Pregnant woman, Perinatal care, Length of stay, Legislation as topic

## Abstract

**Objective:**

To evaluate the impact of São Paulo State Law n° 17.137/2019 on the cesarean section rate at a public secondary-level maternity hospital and to analyze predictive factors and complications associated with cesarean under request. This law was enacted to allow pregnant women in São Paulo to request a cesarean section without medical indication.

**Methods:**

This retrospective study analyzed medical records of pregnant women ≥ 39 weeks gestation attended at the Ribeirão Preto Women's Health Reference Center (CRSMRP-Mater). Two groups were evaluated: 1,999 patients before the law (July 2018–July 2019) and 3,207 after its implementation (August 2019–July 2021, excluding the suspension period). Descriptive and analytical statistical methods were applied.

**Results:**

The overall cesarean rates increased significantly from 23.6% to 27.7% (p < 0.01), with 15,1% of cesareans during the law period being under maternal request (134 patients). A previous cesarean was the only factor significantly associated with electing a new cesarean. Hospital length of stay was significantly longer in the law period (p < 0.01), possibly reflecting the increased cesarean rate. No significant differences were observed in maternal or neonatal complications between cesareans under request and those conducted for medical reasons.

**Conclusion:**

São Paulo State Law n°. 17.137/2019 was associated with an increased cesarean rate in CRSMRP-Mater. The findings highlight the need for robust educational approaches and evidence-based obstetric practices to reduce unnecessary elective cesareans.

## Introduction

Although the cesarean section is a surgical procedure intended to reduce maternal and perinatal morbidity and mortality, it presents both immediate and long-term risks. The World Health Organization (WHO) recommends that cesarean rates not exceed 15%, as higher rates have not demonstrated a positive impact on the perinatal mortality rates.^(1)^ However, considering the Brazilian population characteristics, the adjusted cesarean rate recommended by the Brazilian Ministry of Health, based on an instrument developed by WHO, is 25-30%.^(2)^ Despite this, Brazil faces alarming rates, reaching up to 55% in the Unified Health System (SUS) and up to 90% in the private sector, reflecting a real epidemic of surgical births.^(3)^

Medical indications for cesarean delivery include labor dystocia, fetal vitality disorders, cephalopelvic disproportion, abnormal fetal presentations, multiple pregnancies, some infections, certain maternal diseases, and placental disorders, as outlined by the American College of Obstetricians and Gynecologists (ACOG).^(4)^ However, a growing phenomenon is the maternal requested cesarean, driven by factors such as labor pain, anxiety, and previous childbirth experiences.^(5)^ Studies show that fear of childbirth can lead women to demand a cesarean even in the absence of medical indications, increasing the likelihood of unnecessary surgical interventions.^(6–8)^

In this context, São Paulo State Law No. 17.137/2019,^(9)^ which authorizes cesarean sections at the request of pregnant women with at least 39 weeks of pregnancy, has brought to light issues regarding maternal autonomy and healthcare professionals responsibility of informing about the risks and benefits of different delivery routes.^(4)^ Therefore, understanding the factors that lead pregnant women to choose cesarean deliveries is essential, as this choice impacts not only individual health but also the healthcare system as a whole.^(10)^

Thus, this study wanted to analyze the prevalence of cesareans under maternal request, their predictive factors, and obstetric outcomes at a secondary complexity maternity hospital, the Ribeirão Preto's Women Health Reference Center - MATER (CRSMRP-MATER). This analysis was conducted considering the context of the implementation of São Paulo State Law No. 17.137/2019,^(9)^ aiming to evaluate its impact and to contribut to a broader understanding of maternal requested cesareans and their implications for public health.

## Methods

This was a retrospective study including a convenience sample of pregnant women with a gestational age of 39 weeks and beyond assisted at the "Ribeirão Preto Women's Health Reference Center - MATER" (CRSMRP-MATER), between July 1, 2018, and July 31, 2019 (previously to São Paulo State Law No. 17.137/2019 effectiveness), and from August 23, 2019, to July 31, 2021 (after the enactment of the mentioned law), for comparison of cesarean delivery rates. Pregnant women assisted between July 1, 2020, and June 30, 2021, were excluded from the sample due to the law's suspension during this period, since the study aimed to analyze precisely the law's effects. CRSMRP-MATER is a public service with a secondary maternity ward to provide medical care for patients from the Brazilian Unified Health System (SUS), with a monthly average of 180 deliveries.

To analyze predictive factors associated with cesarean sections requested by the parturient, a new sample was studied, including pregnant women with a gestational age of 39 weeks or more, during the law's effectiveness period. Then, patients who had cesarean section upon their request were compared to those who had cesarean due to an obstetric indication.

Data were obtained from electronic medical records of the patients, including information on age, skin color, occupation, marital status, educational level, obstetric history, labor evolution, and delivery outcomes (onset, patient preferences, delivery route, and obstetric outcomes). Data were anonymized and stored securely on the Research Electronic Data Capture (REDCap) platform, hosted by the Medical School of Ribeirao Preto, from the University of Sao Paulo (FMRP-USP), with restricted access to researchers.

The primary outcome evaluated was the change in the cesarean section rate regarding the pre-law and law-period groups. Secondarily, the predictive factors and the complications related to cesarean under request were analyzed within the group during the law period.

Statistical analysis included an exploratory evaluation of data with absolute and relative frequencies for qualitative variables and measures of central tendency and dispersion for quantitative variables. The chi-square test was used for categorical variables and the Student's t-test or Wilcoxon test for continuous variables, depending on the data distribution. All analyses were conducted using SAS software version 9.4.

Since the study used secondary data from medical records without interfering in obstetric management, it was exempt from obtaining informed consent. The study was approved by the Ethics Committee of the Ribeirao Preto Clinical Hospital (HCRP), and by the Research Commission of CRSMRP-MATER 5.439.837 (*Certificado de Apresentação de Apreciação Ética* (CAAE): 58799621.1.0000.5440).

## Results

A total of 5,206 patients were included, with all deliveries performed during the specified period at the hospital and divided into two groups: 1,999 in the pre-law period (all deliveries between July 1, 2018, and July 31, 2019) and 3,207 in the law period (all deliveries between August 23, 2019, and July 31, 2021, excluding the law's suspension period).

In both groups, the median age and parity were 25 years and two pregnancies, respectively. The sociodemographic evaluation revealed a predominance of white patients with an educational level of at least nine years of study, no stable marital relationship, no personal income, and no pre-existing diseases in both groups (Table 1). Despite these similarities, in the pre-law period there were a significantly higher proportion of white patients (p < 0.01), patients without a stable marital relationship (p < 0.01), and those without personal income (p < 0.01).

**Table 1:** Distribution of age, parity, and sociodemographic characteristics of the sample

Variables		Total	Law period	Pre-law period	p-value
			n/total(%) n(%)	n/total(%) n(%)	
Age (years)*		5,203	25.9 ± 6.1	25.6 ± 6.2	0.473
Number of Pregnancies*		5,203	2.1 ± 1.2	2.1 ± 1.2	0.405
Skin color	White	5,187	1,915/3,192(60)	1,384/1,995(69.4)	< 0.01
	Black or brown		1,277/3,192(40)	611/1,995(30.6)	
Educational Level	0 years	5,156	9/3,169(0.3)	6/1,987(0.3)	0.78
	1 to 8 years		859/3,169(27.1)	556/1,987(28)	
	9 years or more		2,301/3,169(72.6)	1,425/1,987(71.7)	
Marital status	Stable partner	5,111	1,157/3,127(37)	530/1,984(26.7)	< 0.01
	No stable partner		1,970/3,127(63)	1,454/1,984(73.3)	
Occupation	With personal income	5,165	1,282/3,174(40.4)	731/1,991(36.4)	< 0.01
	Without personal income		1,892/3,174(59.6)	1,260/1,991(63.3)	

* Continuous variables expressed in mean ± standard deviation and compared with t-student or Wilcoxon test. Categorical variable expressed in absolute and relative frequencies, and compared with the Chi-squared test

Both groups were homogeneous regarding the presence of a previous cesarean section, prenatal care, number of fetuses (all were single pregnancies), birth weight, and vitality at birth (Table 2). Although both groups predominantly consisted of healthy pregnant women, the proportion of pregnant women with comorbidities, particularly hypertension and diabetes, was significantly higher in the law period (p < 0.01). Regarding the primary outcome, there was a significant increase in the cesarean rate of the institution, rising from 23.6% during the pre-law period to 27.7% in the law period (p < 0.01). As for the main complications evaluated, the rate of postpartum hemorrhage was similar between the groups, and also was the need for re-hospitalization, which occurred in less than 3.0% of both groups, with most of these cases unrelated to childbirth (e.g., re-hospitalization for newborn phototherapy). The length of hospitalization was significantly longer in the law period (p < 0.01), possibly related to the higher proportion of cesareans in this group (p < 0.01).

**Table 2:** Distribution of obstetric and perinatal outcomes

Variables		Law period	Pre-law period	p-value
		n/total(%) n(%)	n/total(%) n(%)	
Previous cesarean	Yes	529/3,207(16.5)	325/1,996(16.3)	0.84
	No	2,678/3,207(83.5)	1,671/1,996(83.7)	
Diseases during pregnancy	Diabetes	234/3,166(7.4)	62/1,995(3.1)	< 0.01
	Hypertensive disorder	304/3,166(9.6)	44/1,995(2.2)	
	Other	434/3,166(13.7)	443/1,995(22.2)	
	No	2194/3,166(69.3)	1,446/1,995(72.5)	
Prenatal care	Yes	2,874/2,994(96)	1,880/1,940(96.9)	0.11
	No	120/2,994(4)	60/1,940(3.1)	
Labor onset	Spontaneous	2,070/3,205(64.6)	1,335/1,992(67)	< 0.01
	Induced	872/3,205(27.2)	567/1,992(28.5)	
	Resolution by cesarean	263/3,205(8.2)	90/1,992(4.5)	
Pharmacological labor analgesia	Yes	1,373/3,207(42.8)	790/1,991(39.7)	0.03
	No	1,834/3,207(57.2)	1,201/1,991(60.3)	
Delivery route	Cesarean before labor	202/3,206(6.3)	134/1,997(6.7)	< 0.01
	Cesarean after labor onset	548/3,206(17.1)	293/1,997(14.7)	
	Elective cesarean	138/3,206(4.3)	44/1,997(2.2)	
	Vaginal delivery	2,318/3,206(72.3)	1,526/1,997(76.4)	
Indication for cesarean	Under request	37/888(4.2)	0/471(0)	< 0.01
	Other indication	851/888(95.8)	471/471(100)	
Birth weight	≤ 2500g	35/3,166(1.1)	40/1,986(2)	0.05
	2501 a 3999g	2,935/3,166(92.7)	1,827/1,986(92)	
	> 3999g	196/3,166(6.2)	119/1,986(6)	
Vitality at birth	Alive	3,193/3,196(99.9)	1,993/1,993(100)	0.11
	Deceased	3/3,196(0,1)	0/1,993(0)	
Apgar score in 5th minute	0 to 3	6/3,068(0.2)	2/1,976(0.1)	0.63
	4 to 7	61/3,068(2)	34/1,976(1.7)	
	8 to 10	3,001/3,068(97.8)	1,940/1,976(98.2)	
Postpartum hemorrhage	Yes	346/3,204(10.8)	168/1,995(8.4)	< 0.01
	No	2,858/3,204(89.2)	1,827/1,995(91.6)	
Hospitalization time	≤ 2 days	2,165/3,203(67.6)	1,460/1,995(73.2)	< 0.01
	> 2 days	1,038/3,203(32.4)	535/1,995(26.8)	
Re-hospitalization	Yes	83/3,204(2.6)	50/1,995(2.5)	0.76
	No	3,121/3,204(97.4)	1,945/1,995(97.5)	
Reason for Re-hospitalization	Related to childbirth	18/83(21.7)	9/44(20.4)	0.87
	Not related to childbirth	65/83(78.3)	35/44(79.6)	

A total of 134 patients in the law period had cesarean deliveries under request, representing 4,2% of all deliveries and 15,1% of all cesareans done during this period. In subsequent analyses, this subgroup of women with cesarean deliveries under request was compared to women with medically indicated cesarean deliveries. Within these comparisons, no sociodemographic differences were observed between the subgroups, and the sample characteristics remained consistent: a predominance of white women, with at least nine years of education, no stable marital relationship, and no personal income (Table 3).

**Table 3:** Sociodemographic characteristics of women who underwent cesarean section during the law period

Variables		Cesarean under request	Medically indicated cesarean	p-value
Skin color	White	82/132(62.1)	459/749(61.3)	0.85
	Brown or Black	50/132(37.9)	280/749(37.8)	
Educational level	0 years	0/133(0)	3/743(0.1)	0.51
	1 to 8 years	24/133(18.1)	163/743(22.2)	
	9 to 13 years	109/133(81.9)	577/743(77.7)	
Marital status	Stable partner	60/133(45.1)	270/738(36.6)	0.06
	No stable partner	73/133(54.9)	468/738(63.4)	
Occupation	With personal income	61/134(45.5)	361/742(48.6)	0.50
	Without personal income	73/134(54.5)	381/742(51.4)	

During the evaluation of obstetric and perinatal outcomes during the law period (Table 4), it was observed that both subgroups (cesarean by maternal request and cesarean by medical indication) were similar in terms of healthy pregnant women predominance, and good adherence to prenatal care. No differences were found regarding the rate of intrapartum pharmacological analgesia between the subgroups. However, there was a higher occurrence of cesarean deliveries before the onset of labor in the maternal requested cesarean subgroup (p < 0.01), as well as a predominance of women with one or more previous cesareans (p < 0.01). Finally, there were no differences between these subgroups regarding neonatal vitality, length of hospitalization, and perinatal complications.

**Table 4:** Distribution of obstetric and perinatal outcomes for women in the law period

Variables		Cesarean by maternal request	Cesarean by medical indication	p-value
Previous cesarean	Yes	70/134(52.2)	187/754(24.8)	< 0.01
	No	64/134(47.8)	567/754(75.2)	
Diseases during pregnancy	Diabetes	16/130(12.3)	63/746(8.6)	0.48
	Hypertensive disorder	14/130(10.8)	101/746(13.5)	
	Other	16/130(12.3)	94/746(12.6)	
	No	84/130(64.6)	488/746(65.4)	
Prenatal care	Yes	127/130(97.7)	697/710(98.17)	0.71
	No	3/130(2.3)	13/710(1.83)	
Labor onset	Spontaneous	57/134(42.6)	207/753(27.5)	< 0.01
	Induced	44/134(32.8)	239/753(31.7)	
	Resolution by Cesarean	33/134(24.6)	307/753(40.8)	
Pre-labor analgesia	Yes	65/134(48.5)	373/754(49.5)	0.84
	No	69/134(51.5)	381/754(50.5)	
Vitality at birth	Alive	133/134(99.2)	751/753(99.7)	0.38
	Deceased	1/134(0.8)	2/753(0.3)	
Apgar score at 5th minute	0 to 3	0/124(0)	4/733(0.5)	0.10
	4 to 7	0/124(0)	22/733(3)	
	8 to 10	124/124(100)	707/733(96.5)	
Postpartum hemorrhage	Yes	22/134(16.4)	114/753(15.1)	0.71
	No	112/134(83.6)	639/753(84.9)	
Fever postpartum	Yes	1/134(0.7)	17/751(2.3)	0.25
	No	133/134(99.3)	734/751(97.7)	
Hospitalization time	≤ 2 days	77/134(57.5)	369/752(49.1)	0.07
	> 2 days	57/134(42.5)	383/752(50.9)	
Re-hospitalization	Yes	6/134(4.5)	27/752(3.6)	0.62
	No	128/134(95.5)	725/752(96.4)	
Reason for Re-hospitalization	Related to childbirth	3/6(50)	15/27(55.6)	0.81
	Not related to childbirth	3/6(50)	12/27(44.4)	

## Discussion

Although it was expected, the present study objectively demonstrated that the implementation of São Paulo State Law No. 17.137/2019 coincided with a significant increase in the overall rate of cesareans at CRSMRP-MATER. Among the women who had cesarean deliveries under request, the majority (52.2%) had a previous cesarean, which was the only factor identified in this study as being associated with the maternal request for a new cesarean delivery. This finding is consistent with other studies showing that younger women and those with a history of cesarean delivery are more likely to choose this delivery route again.^(3)^ Nonetheless, the rate of cesareans under maternal request at CRSMRP-Mater in 2022 was relatively low, accounting for only 4.5% of all cesarean deliveries.

When it comes to maternal preferred delivery route, cesarean is chosen by 27% of the Brazilian pregnant women in the public sector, and by 44% in the private sector.^(3)^ All over the world, reasons for requesting a cesarean section include labor pain, anxiety about fetal distress or death, fear of vaginal delivery, urinary incontinence, pelvic floor and vaginal tearing, medical suggestion, timing of delivery, negative previous birth experiences, previous infertility, anxiety about gynecological exams, fear of losing control, desire to avoid prolonged labor, anxiety due to lack of support from the healthcare providers, fear of fecal aspects, emotional factors, estimated fetal weight, and abnormal prenatal tests.^(5)^ It is important to note that when fear of childbirth is not addressed, the likelihood of requesting a cesarean section may increase up to 5.2 times.^(11,12)^ This can lead to unnecessary surgery and expose the patient to avoidable risks.^(13,14)^

In Brazil, the main factors justifying the preference for cesarean section are maternal convenience and fear of pain, with the latter being the primary cause of fear of childbirth as a whole.^(2,15)^ A contributing factor is also the lack of information among Brazilian pregnant women about delivery routes, which hinders their understanding of the risks and benefits of both cesarean and vaginal birth.^(11)^ Additionally, there has been historically a disparity in obstetric care between the public and private sectors. In the context of private healthcare, cesarean sections are predominantly performed, often due to maternal desire. In contrast, although the cesarean rates in the public health system are high, they are more restrained, as parturients did not have full autonomy to choose their mode of delivery until now.^(16)^ In this sense, the justification for São Paulo State Law No. 17.137/2019^(9)^ is based on the goal of providing greater autonomy to pregnant women, aiming to promote horizontal relationships between doctors and patients. Although this is a valid objective, the argument behind this law did not consider aspects related to the risks of surgical complications and the impact of unnecessary cesarean sections on the reproductive future of patients, as literature shows higher risks of severe maternal morbidity, maternal mortality, and neonatal respiratory morbidity related to the cesarean, as well placental abnormalities in subsequent pregnancies.^(17–20)^ Finally, while the law assumes that the costs of vaginal and cesarean deliveries are similar in the healthcare system, the literature demonstrates that the average cost of a cesarean section is 32% higher than the cost of a vaginal birth, including the length of stay in the maternity ward after each procedure.^(2)^

The initial revocation of São Paulo State Law No. 17.137/2019^(9)^ and its subsequent reinstatement by the Federal Supreme Court reveals the complexity of the ethical and legal considerations surrounding its implementation. While the law was enacted to safeguard a woman's right to exercise full autonomy in selecting her mode of delivery, this decision must be informed and supported by robust scientific evidence exploring the safety and risks associated with both options. To achieve this, health education must be prioritized to provide pregnant women with clear and comprehensive information regarding the implications of their choices.^(21,22)^

In this sense, a study conducted at another institution in the city of Ribeirão Preto-SP revealed that 48% of births were cesarean deliveries, of which 44.6% were performed due to maternal request.^(23)^ This contrasts with the rates found in the present study, which were 27.7% and 15.1%, respectively. It is noteworthy that CRSMRP-MATER has been actively engaged in health education and awareness for pregnant women, especially those who express a desire for cesarean during prenatal care. The goal of this initiative is to understand the reasons behind this choice, to equip women with evidence-based information translated into accessible language, enabling them to identify and deconstruct unfounded fears and expectations, and consequently to make truly informed decisions. As a result, many patients end up renouncing their initial desire, which contributes to the reduction of cesarean rates without obstetric reason.

Nonetheless, medical factors must also be considered when analyzing cesarean rates. The presence of gestational diseases was significantly higher in the group that gave birth after the enactment of the law, the one with a higher proportion of cesarean deliveries, raising the question of whether these conditions influenced the decision to undergo the surgery. Literature suggests that the presence of comorbidities may increase the perceived risk associated with vaginal delivery, leading to a preference for cesarean delivery.^(4)^ However, the decision for cesarean delivery in the absence of a medical indication exposes pregnant women to unnecessary risks, such as surgical complications, longer hospitalization, and increased maternal and neonatal morbidity.

In parallel, while the group from the law period had higher rates of pharmacological analgesia and cesarean deliveries than the one from the period before the law, the literature is clear in demonstrating that the use of pharmacological analgesia does not lead to increased cesarean rates.^(24)^ Thus, considering that the institution provides pharmacological analgesia freely to all parturients, even before the enactment of the law, and that no differences were observed in analgesia rates between patients with maternal requested cesareans and those with medically indicated cesareans, the higher cesarean rate in the law period can be better explained by the law itself, that allowed cesarean delivery upon request.

Finally, it is known that cesarean delivery is linked to short- and long-term risks for both mothers and newborns, including higher maternal morbidity, neonatal respiratory issues, and NICU admissions.^(25,26)^ Long-term maternal risks involve complications in future pregnancies, while children face increased chances of asthma and obesity.^(27,28)^ Despite of this, no differences were observed in the rates of short-term postpartum complications studied between the maternal requested cesarean subgroup and the medically indicated cesarean subgroup in this sample.

Despite the relevance of the findings, it is essential to acknowledge the strengths and limitations of the present study. Among the strengths of this study is the use of a comprehensive and representative institutional database, with detailed information on deliveries performed over one year at a public secondary maternity hospital. This enabled the identification of relevant patterns associated with cesarean under request and increasing in the cesarean rate of the studied healthcare service.

However, the study has its limitations, including the retrospective design and reliance on the accuracy of medical records. Furthermore, as it was conducted in a single center, the findings may not be generalizable to other populations or hospital settings.

## Conclusion

The analysis of the impact of São Paulo State Law No. 17.137/2019 in a secondary complexity maternity hospital in São Paulo revealed an alarming increase in cesarean rates. Although no increment in complications related to maternal-requested cesareans was observed compared to medically indicated cesareans, the study demonstrated higher rates of postpartum hemorrhage and longer hospitalization times in the law period, with a predominance of cesareans compared to vaginal births. Given the complexity of the issue, it is essential to gain a better understanding of the surgical birth choice and its impact on the healthcare system, which will be possible through future studies, particularly prospective ones. Thus, only with more information will healthcare teams be able to define better strategies to attempt to reduce cesarean birth rates in Brazil.
